# European expert guidance on management of sleep onset insomnia and melatonin use in typically developing children

**DOI:** 10.1007/s00431-024-05556-w

**Published:** 2024-04-16

**Authors:** Oliviero Bruni, Maria Breda, Lino Nobili, Ingo Fietze, Oscar Ramon Sans Capdevila, Claude Gronfier

**Affiliations:** 1 Department of Developmental and Social Psychology, Sapienza University of Rome, Rome, Italy; 2 Department of Psychology, Sapienza University of Rome, Rome, Italy; 3 Department of Neurosciences, Rehabilitation, Ophthalmology, Genetics and Maternal and Child Health, University of Genoa, Genoa, Italy; 4 Child Neurology and Psychiatry, Istituto G. Gaslini, Genoa, Italy; 5 Interdisciplinary Center of Sleep Medicine, Charité-Universitätsmedizin Berlin, Berlin, Germany; 6 Department of Medicine, Southwest Medical University Affiliated Zigong Hospital, Zigong, Sichuan China; 7 Sleep Unit at the Sant Joan de Déu Children’s Hospital in Barcelona, Barcelona, Spain; 8 International University of Catalonia (UIC), Barcelona, Spain; 9 Lyon Neuroscience Research Center (CRNL), Neurocampus, Waking Team, Inserm UMRS 1028, CNRS UMR 5292, Université Claude Bernard Lyon 1, Université de Lyon, 69000 Lyon, France

**Keywords:** Insomnia, Melatonin, Children, Adolescents, Pediatricians

## Abstract

Sleeping problems are prevalent among children and adolescents, often leading to frequent consultations with pediatricians. While cognitive-behavioral therapy has shown effectiveness, especially in the short term, there is a lack of globally endorsed guidelines for the use of pharmaceuticals or over-the-counter remedies in managing sleep onset insomnia. An expert panel of pediatric sleep specialists and chronobiologists met in October 2023 to develop practical recommendations for pediatricians on the management of sleep onset insomnia in typically developing children. When sleep onset insomnia is present in otherwise healthy children, the management should follow a stepwise approach. Practical sleep hygiene indications and adaptive bedtime routine, followed by behavioral therapies, must be the first step. When these measures are not effective, low-dose melatonin, administered 30–60 min before bedtime, might be helpful in children over 2 years old. Melatonin use should be monitored by pediatricians to evaluate the efficacy as well as the presence of adverse effects.

*    Conclusion*: Low-dose melatonin is a useful strategy for managing sleep onset insomnia in healthy children who have not improved or have responded insufficiently to sleep hygiene and behavioral interventions.

**Supplementary Information:**

The online version contains supplementary material available at 10.1007/s00431-024-05556-w.

**Table:** 

**What is Known:** *• Pediatric insomnia is a common disorder and impacts children's emotional, behavioral, and cognitive functioning, as well as parents' sleep and daytime functioning.* *• There is no consensus in Europe on the management of sleep onset insomnia and the use of melatonin in typically developing children.*	
**What is New:** *• A group of European experts has compiled a set of recommendations for the management of insomnia, developing a step-by-step approach.* *• Sleep hygiene, behavioral strategies, and finally low-dose melatonin represent valid strategies for managing sleep onset insomnia.*	

## Introduction and problem statement

Sleep disorders are very common in children and adolescents and represent a frequent reason for pediatric consultation [1]. Although a relatively small number of children suffer from intrinsic sleep disorders that require specialist medical care (such as sleep apnea, restless legs syndrome, or narcolepsy) and in a more considerable number of cases sleep problems occur in children suffering from chronic health conditions or mental distress (e.g., depression and anxiety), most children experience insomnia and problems falling asleep linked to chronic insomnia, evening chronotype, and circadian misalignment during changes in daily routine, schedule, or stressful situations such as exams and competitions. These situations might be difficult to manage since most pediatricians and nurse practitioners are not trained about behavioral sleep problems [2, 3].

The International Classification of Sleep Disorders, ICSD-3 [4] has included the “paediatric insomnia” into a single entity of the chronic insomnia disorder; however, it still includes three subtypes: sleep-onset association insomnia, limit-setting insomnia, and a combined type.

The chronic insomnia disorder may be diagnosed as early as 6 months of age, and its occurrence in the first years of life is very high. In European countries, the prevalence of pediatric insomnia is estimated to be around 15–30% in toddlers (3–5 years), 11–15% in school age (6–12 years), and 20–30% in adolescents [5, 6]. These percentages are quite alarming if we consider the consequences of insufficient sleep in these particularly vulnerable age groups. Insufficient sleep could be the result of inadequate sleep duration, poor sleep quality, or both [7]. Sleep quality refers to the subjective indices of how sleep is experienced including the feeling of being rested when waking up and satisfaction with sleep [8], but this feeling is not reliably reported by children [9, 10]. While the impact of sleep quality on children functioning seems to be more important than that of sleep quantity [8], there is little research that focuses specifically on the quality of sleep in children, due to their limited expressive capacity and the difficulty to perform polysomnography in children suffering from insomnia.

**Fig. 1:**
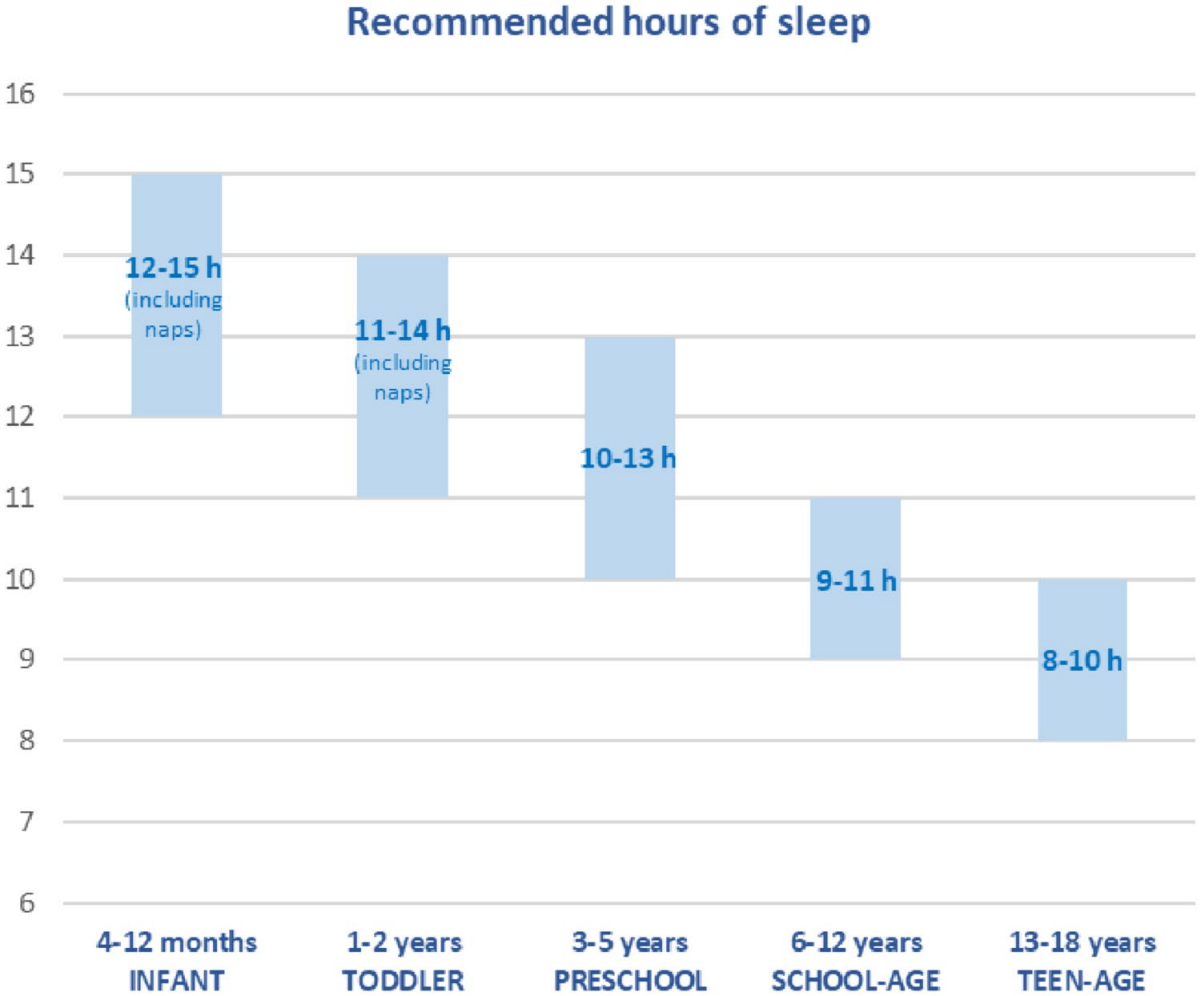
Hours of sleep for children and adolescents recommended by the National Sleep Foundation. Modified from Hirshkowitz et al. [11]

Figure 1 shows the recommendations developed by the National Sleep Foundation for sleep duration within a 24-h cycle for children and adolescents [11]. Even 30–60 min less of sleep per night significantly reduces children’s health-related quality of life [12] and impacts children’s emotional, behavioral, and cognitive functioning [13]. Insomnia has an impact on brain activity and connectivity, resulting in a wide range of disruptions in human cognition and affect [14]. This seems especially valid for insomnia that persists from childhood to adolescence [15], which seems to happen in 56% of cases [16]. The peak period of incidence of insomnia in childhood constitutes, in fact, a period of particular susceptibility of the prefrontal cortex, responsible for executive functioning and regulatory behavior. Besides poor sleep, other factors such as mental health, stress, and abuse might have detrimental effects on the prefrontal cortex, affecting the development of executive functioning and regulatory behavior, with important long-term implications [17–19].

The effects of inadequate sleep and poor sleep quality can therefore extend from impaired neurocognitive development, expressed by thought problems and difficulties in crystallized intelligence, to behavioral and emotional problems, as externalizing behaviors, depression, and anxiety [16, 20, 21]. Clearly, insufficient sleep may be both a cause and a consequence of psychopathologic conditions; this is confirmed by the fact that sleep disorders in adolescence have been bidirectionally linked to mental health problems, leading to a vicious unhealthy circle [22].

Poor sleep impacts not only mental health and cognitive functions already in the first years of life [23] but also children’s global health. In adolescents, insufficient sleep duration has been associated with higher blood pressure [24], insulin resistance [25], obesity [26], and increased cardiometabolic risk [27]. In children, it has been linked with excessive screen time, unhealthy dietary habits, and obesity [28, 29].

The effects of a child’s disturbed sleep do not only affect physical and psychosocial health but expand to the whole family’s functioning and well-being [30, 31]. Sleep problems in children and adolescents are associated with poor sleep quality, stress, fatigue, and reduced physical health in parents [30, 32]. Insomnia in children is also associated with an increased risk of infant/child abuse by exhausted parents [33–36]. Reciprocal relations between marital or familiar conflict and sleep disruptions in children and adolescents have been highlighted [37, 38].

The management and treatment of sleep problems in childhood and adolescence are therefore fundamental, as it may lead to an improvement not only in the child/adolescent’s sleep but also in the parents’ sleep and daytime functioning.

Cognitive behavioral therapy for insomnia (CBT-I) is the first-line treatment for adults, and existing studies show promising effects also for children and adolescents [39]. Although there is considerable evidence of the effectiveness of CBT-I, at least in the short term, there are currently no internationally approved recommendations for the pharmacological or over-the-counter (OTC) management of sleep onset or sleep maintenance insomnia in children.

Study findings indicate developmental shifts in the prevalence of sleep behaviors and sleep problems, as well as how caregivers characterize sleep problems by child’s age. Waking overnight is the most common sleep behavior during infancy and early childhood, reflecting normative patterns of child sleep consolidation. Waking overnight decreases with age and after the age of 4 years, the main complaint is the difficulty sleeping independently and namely sleep onset insomnia [40]. These problems correspond with manifestations of insomnia disorder in early development, including problematic sleep onset associations (e.g., feeding or parental presence at bedtime) and the common emergence of nighttime fears and bedtime refusal in early childhood. Unlike in earlier development, difficulty falling asleep is more prevalent between ages 6 and 11 years [40]. Based on this evidence, the more prevalent problem in preschool and school children is the sleep onset insomnia.

### The role of melatonin in management of sleep onset insomnia in children

Melatonin is an endogenous hormone, primarily synthesized by the pineal gland at night. Its rhythmic secretion is regulated by the circadian clock located in the suprachiasmatic nucleus in the hypothalamus which is influenced by the daily alternation of darkness and light. It is also influenced by numerous signals originating from various sources and brain structures [41], and it is strongly inhibited by light, even at relatively low intensities [42, 43]. Through its chronobiological effect, melatonin plays a key role in regulating the sleep–wake cycle, but it also has antioxidant, anti-inflammatory, and free radical scavenging properties [44]. The effect of melatonin on sleep is believed to be a consequence of its actions on MT2 and MT1 receptors, which are involved in NREM and REM sleep, respectively [45].

Melatonin is considered a “dietary supplement” by the FDA and a “natural health product” by the Health Products and Food Branch in Canada [46]. Low doses are also considered food supplement in most of the European countries [47]. In the United States, melatonin is the second most commonly used product among all children evaluated in a US National Health Interview Survey [48]. Caregivers commonly associate melatonin with “naturalness” and “safety,” and for many of them, melatonin treatment is a “life-saver” and “like a miracle”, “chang[ing] the life of the entire family” [49].

Although there are several data from scientific literature [50, 51] on the efficacy and safety of long-term melatonin for insomnia in children and adolescents with neurodevelopmental disorders, few data are available in the typically developing pediatric population. A recent systematic review and meta-analysis on melatonin use in children with idiopathic chronic sleep onset insomnia [52] found a moderate increase in total sleep time of 30 min and a moderate decrease in sleep latency of 18 min. The studies reviewed did not provide information on serious adverse events associated with melatonin use; there were only non-serious adverse events, such as headaches, nausea, red eyes, drowsiness, changes in mood and cognition, and gastrointestinal problems. Another recent systematic review on short-term and long-term adverse effects of melatonin in children and adolescents with chronic insomnia [53] found that melatonin treatment was not associated with serious adverse events and reported some level of uncertainty regarding the extent to which melatonin leads to non-serious adverse events. A recent meta-analysis study showed that in non-comorbid insomnia and comorbid insomnia melatonin had a significant effect on sleep onset latency and total sleep time in the children and adolescents group [54].

Although there are no internationally accepted guidelines on use of melatonin in normally developing children with sleep onset insomnia, management algorithms have been developed and published independently by experts in Canada [55] and Spain [56], along with a consensus paper produced during a conference in Rome [57] and a clinical recommendation based on systematic review and meta-analysis [52]. All these recommendations are very similar in the steps for treating sleep onset insomnia: the Canadian one includes sleep hygiene as first step and CBT-I with or without melatonin as second step [55]; the Spanish one suggests sleep hygiene and CBT-I as first step then addition of melatonin [56]; the consensus in Rome suggested to use melatonin as sleep inducer or chronobiotic [57]; the systematic review provided no algorithms but formulated the recommendation for melatonin only if sleep hygiene and non-pharmacological interventions have proven inadequate [52].

While recommendations from previous studies were directed to non-European countries, single European country, or to all pediatric population (typically developed children and not), this paper aims to support European primary care pediatricians in their clinical practice summarizing the views expressed by European experts in a recent Consensus Panel meeting, convened in October 2023, on the management of sleep onset insomnia and the use of melatonin in normally developing children.

## Methods

### Selection of the experts

To identify potential experts, an initial literature review was conducted to identify European key researchers, practitioners, and thought leaders in the field. Additionally, recommendations were sought from reputable organizations and professional networks related to the study’s topic. These sources provided a pool of candidates who were considered for inclusion in the expert panel. The selection process involved multiple stages. Initially, around 20 identified experts were invited to participate based on their expertise and contributions in the field. They were asked to submit their credentials, including their educational background, professional experience, and relevant publications. Once the initial pool of potential experts was established, a selection committee, comprising individuals with expertise in the subject matter and research methodology, reviewed the credentials of each candidate. The committee assessed their expertise, experience, and diversity in terms of geographical location and professional background. The aim was to ensure a balanced representation of perspectives and to minimize bias. Following the committee’s evaluation, the final panel of experts was selected. Invitations were sent to the chosen experts.

### Nominal group technique

For the purpose of the study, we used the nominal group technique (NGT) that is a structured method for group brainstorming that consists of 5 steps: (1) introduction, (2) silent idea generation, (3) idea sharing, (4) group discussion, and (5) voting. The nominal group technique can be used by small groups to reach consensus on the identification of key problems or in the development of solutions that can be tested using rapid-change cycles.

Before, the meeting the group leader (O.B) sent a document that clarified the objective of the meeting and outlined individual roles and the voting method. Each participant was asked to collect and read the main important papers on the melatonin use in typically developing children, with the priority for topical and systematic reviews and meta-analysis. Specific questions for the purpose of the meeting were prepared and presented by the group leader. During the meeting, the group leader welcomed the participants and explained to them the purpose and procedure of the meeting. After step 2, the group leader collected the different ideas and opinions of the participants on the role of melatonin in typically developing children and shared with the others. The group discussion was devoted to the clarification of any disagreements recording the differences of opinion of the participants. The group leader finally assembled the statements and asked the participants to vote or rank. Based on the votes, a final consensus was reached.

## Recommendations

Sleep problems are frequently underreported by parents if the issue does not create significant negative consequences on family functioning. Sleep and circadian rhythmicity should be assessed by primary care pediatricians during the periodic child health visit, as recommended also by the American Academy of Pediatrics [58]. A thorough assessment by pediatricians should encompass an examination of the child’s current sleep patterns, typical daytime and nighttime sleep duration and its regularity/variability, sleep/wake schedule (timing of sleep within the 24-h day and its regularity/variability), evening routines prior to bedtime, and daytime symptoms (sleepiness, alertness level, behavioral disturbances, etc.).

It is important to check that the child’s sleep duration respects the recommendations exposed in Fig. 1 [11] and to ensure that the quality of sleep is adequate by asking questions about daytime functioning and sleepiness and about subjective assessment of “good” or “poor” sleep and investigating the presence of any abnormal behavior during the night (e.g., excessive movements, bruxism, snoring, open mouth breathing, night terrors, and enuresis).

**Fig. 2:**
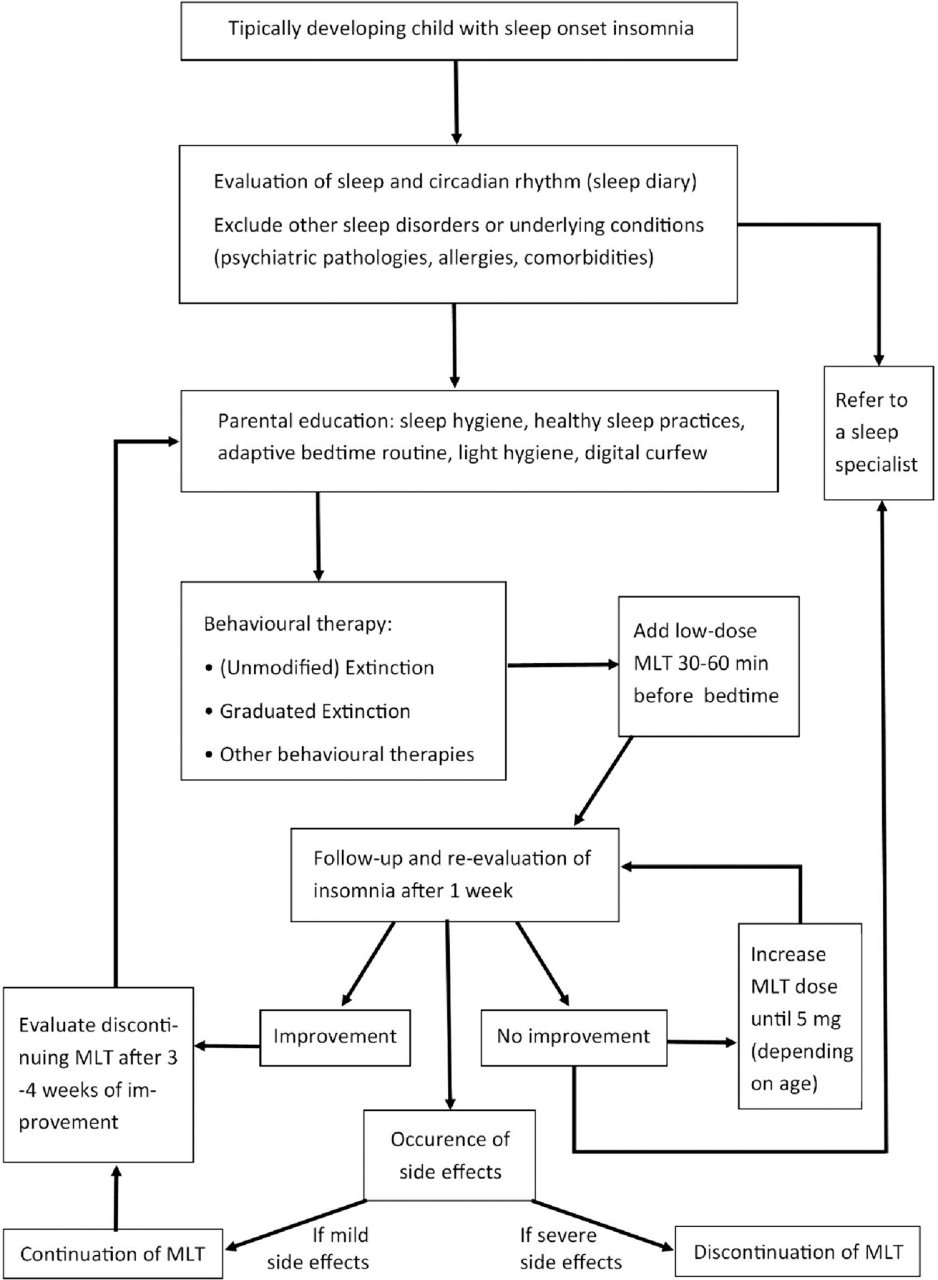
Flowchart for management of sleep onset insomnia. MLT, melatonin

When the sleep problem occurs in comorbidity with other health problems (chronic diseases, psychiatric, or neurological disorders), comorbidity should be assessed and treated separately. When sleep onset insomnia is present in otherwise healthy children, the management should follow a stepwise approach. A simple flowchart is exposed in Fig. 2. The first step should be a comprehensive evaluation of the child’s sleep pattern, sleeping arrangement, bedtime routine, and parental behaviors and responses to the child both at bedtime and after night waking. This is often best achieved using a sleep diary, where parents record the child’s daily sleep behaviors over an extended period of time (usually around 2 weeks).

Pediatricians should inquire about the presence of other sleep disorders (e.g., sleep apnea, narcolepsy, or restless legs syndrome); if suspected, they should request a consultation with a sleep specialist. Furthermore, if there are other underlying conditions that can explain the sleep problem, such as allergies, comorbidities, and potential factors causing pain (e.g., ear infections and reflux), or psychiatric diseases, the healthcare provider should treat the underlying condition or, if necessary, request a consultation with a specialist. After having excluded other possible causes of sleep onset insomnia, pediatricians should manage the problem through parental education on sleep hygiene, healthy sleep practices, and adaptive bedtime routines. Practical sleep hygiene indications to be given to parents are reported in Table 1. Since the role of circadian synchronization is crucial, particular attention should be given to light exposure. Daily sunlight/natural exposure, especially in the morning but also throughout the entire day, and a lowering of light intensity before bedtime permit the proper synchronization of the circadian clock and favor a quicker sleep onset after bedtime. Bedroom should be free from screens and bright light sources, and a “digital curfew” should be observed at least 1–2 h before bedtime (no electronics/screens). If a child requests a nightlight, choose a low light intensity and orange/red light and place it as far as possible from the head of the bed.

**Table 1:** Practical sleep hygiene advices

- Respect the recommended sleep duration for age
- Promote exposure to natural light (sunlight) and physical activity during the day, it helps circadian synchronization
- Remove screens or other light sources in the bedroom before bedtime, if the child requests a nightlight, choose a low light intensity and orange/red light
- Turn off screens 1–2 h before bedtime (digital curfew)
- Create an adaptive bedtime routine, tailored to the individual child and family
- Put the child to bed when he/she is still awake and then leave the child’s bedroom
- Promote stability of the sleep/wake cycle during the week and maintain similar sleep/wake times and bedtime routines on weekdays and weekends
- After a specific age ranging from 3 to 5 years, limit naps early in the afternoon to allow adequate sleep pressure to accumulate by bedtime
- Pay attention to a diet that promotes sleep (prefer a Mediterranean diet; avoid soft drinks, fast food, and snacks instead of meals; and do not skip breakfast)
- Daytime exercise promotes sleep but not too close to bedtime (no exercise within the last 2 h before bedtime)

Attention should also be paid to children’s diet. Following a Mediterranean diet and consuming foods abundant in fiber, fruits, vegetables, and anti-inflammatory nutrients, while minimizing intake of saturated fats, appears to enhance the quality of sleep [59]. Even in toddlers, an increased intake of soft drinks, snacks, and fast foods is linked to shorter, more fragmented sleep patterns, while higher consumption of vegetables is correlated with a more stable sleep [60]. Skipping breakfast, consuming late-night snacks, or substituting meals with snacks are linked to lower overall sleep quality in students [61, 62].

Creating an adaptive bedtime routine should always be recommended as a key factor in the promotion of not only healthy sleep but also of child development and well-being as well as family functioning and caregiver–child bonding [63]. Adaptive bedtime routine includes several activities (such as reading, singing/lullabies, quiet games, bathing and/or brushing teeth, massaging, and cuddling) before lights out. Choosing adaptive bedtime activities, tailored to the individual child and family and to the cultural environment, can promote nurturing care [63] and help the child relax and feel ready for sleep. Many families have a bedtime routine but find difficulties in using it regularly or in having the same bedtime routine on weekdays and weekends [64, 65]. Stability in bedtime routines and sleep/wake time is important to ensure consolidation of sleep/wake rhythmicity and sleep quality [66]. As a conclusion of the bedtime routine, parents should put the child to bed when he/she is still awake, briefly comfort the child, turn the lights out, and leave the child’s bedroom. Worse sleep patterns (reduced night sleep duration and night awakenings) have been associated to the presence of parents while the child is falling asleep, especially if there is active physical comforting [67–69] It is important to promote the development of independence and self-regulation at bedtime and during night awakenings. To help the child to self-sooth, few familiar objects can be placed in child’s bed (avoiding plushies or dangerous objects) [70].

When sleep hygiene and changes in bedtime routines are inefficient, behavioral therapies are recommended. The most used behavioral therapies are unmodified and graduated extinction. Although effective, parental resistance remains the biggest obstacle to these approaches, most of them find extinction too difficult and stressful to implement [71], perhaps due to low parental cry tolerance and high infant distress-attribution cognitions [72]. When behavioral therapies alone are not resolutive or there is parental resistance in implementing them, low-dosage melatonin could be used in combination. Melatonin supplementation can be administered starting with a minimal dose of 0.5 mg, 30–60 min before desired bedtime. Parents should be informed regarding potential adverse events of melatonin use and lack of long-term safety data. They should also be advised to consult with a healthcare professional before using melatonin in children and not use for longer than 14 days without doctor’s recommendation. The bedtime should ensure the recommended sleep duration for the child and therefore depend on his/her age; timing should not be based on the wishes of the child or the parents. In case melatonin is inefficient after 1 week, dosage can be increased step-by-step/gradually, by 0.5 mg at a time, for a week, up to 5 mg, depending on age. Based on the different consensus in the literature, it seems reasonable to consider a dosage of 0.5 to 1 mg in infants 1 to 3 years of age; 1–2 mg in preschoolers, up to 3 mg in school-age children and up to 5 mg in adolescents [52, 55–57]. When a dose is effective, it is recommended to try a lower dose. If side effects occur, it is indicated to discontinue the administration. Although it has been reported that children who use melatonin are likely to experience non-serious adverse events, pediatricians should be aware that common side effects include headache, nausea, red cheeks, red earlobes, sore/red eyes, fatigue/drowsiness, dizziness, vomiting, influenza symptoms/infections, change in mood/cognition, musculoskeletal pain, and gastrointestinal problems [53]. The use of melatonin should not replace general good sleep practices in terms of sleep routines and good sleep hygiene. If using melatonin, importance must be given to circadian synchronization and light exposure, since the effects of light are stronger than that of melatonin on the circadian system and sleep: reduced light exposure and digital curfew are essential before bedtime. Melatonin should be used for 3–4 weeks to stabilize sleep timing and duration, along with continuous sleep hygiene and behavioral corrections; then, it is suggested to suspend it. In rare cases when these techniques do not help, referral to a sleep specialist may be considered. Also, occasional melatonin use (even for a few days) may be beneficial to help in special occasions like changes in the normal routine, flights, and return from vacation, in order to rapidly normalize the sleep–wake pattern.

While there is a wide array of products available, it is crucial to prioritize melatonin sourced from manufacturers with established, high-quality manufacturing standards. A study examining 31 melatonin-containing products and supplements from the US market and not in European market revealed considerable variability in melatonin content, ranging from − 83% to + 478% of the labeled amount. Additionally, 26% of the tested products in the US market contained serotonin, a biosynthetic precursor of melatonin and neurotransmitter associated with various neurological disorders [73]. It is also of utmost importance that the food supplements for children come in the packaging with child-resistant closure to avoid the risk of accidental exposure. Considerate manufacturers of food supplements must also have diligent post-marketing adverse events monitoring system [74].

## Expert advices

An expert panel of pediatric sleep specialists and chronobiologists elaborated a consensus on the use of melatonin in otherwise healthy children.

Although no specific data are available on the safety of melatonin use in otherwise healthy children or children with neurodevelopmental disabilities under 2 years of age, some evidence showed the efficacy and safety of melatonin in older children and adolescents with sleep onset insomnia or delayed sleep phase syndrome [70, 75–77].

1. Advise parents or caregivers on sleep hygiene measures (including light hygiene, nutrition, and digital curfew measures) and behavioral strategies as a first line approach to improve sleep habits
2. If sleep hygiene and behavioral strategies are not effective, melatonin use is recommended in otherwise healthy children with sleep onset insomnia in association with behavioral strategies
3. Melatonin might be helpful for sleep onset insomnia at least in the short-term use
4. Melatonin for sleep induction should be administered 30–60 min before the desired bedtime
5. Start with a minimal dose of 0.5 mg; if no effect after 1 week and then increase the dose with possible titration to 1 mg or more if needed until a maximum of 5 mg depending on age. Consider a dosage of 0.5 to 1 mg in infants 1 to 3 years of age; 1–2 mg in preschoolers, up to 3 mg in school-age children, and up to 5 mg in adolescents
6. Parents should be informed regarding potential adverse events of melatonin use and lack of long-term safety data and advised to consult with healthcare professional before using melatonin in children and not use for longer than 14 days without doctor’s recommendation
7. Melatonin use should be monitored by pediatricians to evaluate the presence of adverse effects
8. Theoretically, since there are no studies in infants and children, melatonin should be avoided in children below the age of 2 years
9. No relevant adverse effects have been reported in different studies for at least 2 years in children with NDDs and specifically no effect on growth or pubertal development; we could expect the same in neurotypical children

### Supplementary Information

Below is the link to the electronic supplementary material.

Supplementary file1 (DOCX 27 KB)

## Declarations

### Disclaimer

The views expressed in the submitted article are his or her own.
