# Supplementary material for: European expert guidance on management of sleep onset insomnia and melatonin use in typically developing children
Source: Eur J Pediatr. 2024 Apr 16;183(7):2955–64. doi: 10.1007/s00431-024-05556-w (PMC11192690; doi:10.1007/s00431-024-05556-w)
Supplement: Supplementary file 1 — Supplementary file1 (DOCX 27 KB) [file 431_2024_5556_MOESM1_ESM.docx]

**SUPPLEMENTARY MATERIAL**

**DESCRIPTION OF THE PROCESS OF THE NOMINAL GROUP TECHNIQUE**

1. **Introduction of the topic and points to be discussed**
2. **Silent idea generation**
3. **Idea sharing from each of the participants**
4. **Group discussion**

Discussion 1: Insomnia in normally developing children, diagnostic challenges, epidemiology and classification.

Discussion 2. Impact of sleep deprivation on children and parents

Discussion 3. Melatonin pathophysiology and specifics of its metabolism in children. Less is which dose is effective ?

Discussion 4: The role of sleep hygiene and cognitive-behavioral treatment of insomnia in children.

Discussion 5: Critical role of pediatrician in providing advice to parents on sleep hygiene measures.

Discussion 6: Addressing safety concerns on melatonin use in children.

Discussion 7: Melatonin as a food supplement, EFSA (European Food Safety Association) view on conditions for use and dosage.

Discussion 8: Align on conditions for use and treatment algorithm of sleep onset insomnia in normally developing children

**After the preliminary discussion of the points, other questions raised and for each of them, after discussion, a consensus was reached by hand vote:**

- *What is the right time to start insomnia management in healthy children?*
  - Start intervention as earlier as possible: insomnia has consequences in early age childhood.
- *When should parents start to get concerned and seek primary care advice? When is the intervention needed?*
  - Commonly parents present late. As soon as they report sleep problems behavioral treatment should be start, after having excluded other sleep problems.
- *Which should be the right approach to evaluate sleep by the pediatrician?*
  - Pediatricians should inquire about the presence of other sleep disorders (e.g., sleep apnea, narcolepsy or restless legs syndrome); if suspected, they should request a consultation with a sleep specialist. Furthermore, if there are other underlying conditions that can explain the sleep problem, such as allergies, comorbidities, potential factors causing pain (e.g., ear infections, reflux, etc.), or psychiatric diseases, the healthcare provider should treat the underlying condition or, if necessary, request a consultation with a specialist.
  - Pediatricians should perform a comprehensive evaluation of the child’s sleep pattern, sleeping arrangement, bedtime routine, and parental behaviors and responses to the child both at bedtime and after night wakings.
  - This is often best achieved using a sleep diary, where parents record the child's daily sleep behaviors over an extended period of time (usually around 2 weeks).

**An additional discussion on the previous topics raised more questions and again a consensus was reached by hand vote:**

*When should pediatricians send patients to specialist?*

After trying 1) behavioral treatment and 2) low dose melatonin

*What is the right starting dose of melatonin in primary care for otherwise healthy children with sleep onset insomnia? Can 0.5 dose be efficacious?*

Dosage in infants, children and adolescents: start with 0.5 mg and increase

*Individual dose titration recommendation.*

Start with the lowest dose possible, if not effective after 1 week increase the dose. If MLT does not work check if they are following the instructions

*What is the right duration of treatment?*

2 weeks -1 month depending on response

*Timing of administration:*

30 minutes before bedtime for sleep onset problems

*Safety of melatonin – short term and long-term safety outcomes:*

Treatment with melatonin is most likely not associated with serious adverse events.

Melatonin supplements at normal doses appear to be safe for most children for short-term use, but there aren’t many studies on children and melatonin.

There’s little information on the long-term effects of melatonin use in children.

No report about rebound effect after discontinuity.

No report of habituation or downregulation

Parents need to ensure safe storage and appropriate use of melatonin supplements.

Use of over-the-counter melatonin might place children and teenagers at risk for accidental or intentional overdose.

Parents should talk to health care professional before giving MLT or any supplement

**As final step we summarized all the points related to the primary care management approach and management steps:**

- providing sleep hygiene and appropriate behavioral measures advise, incl. encouragement of independent falling asleep process, light hygiene, nutrition and digital curfew measures.
- changing family sleep routine: behavioral strategies as a first line approach to improve sleep habits.
- if behavioral measures are not sufficient, low doses of melatonin (starting with 0.5 mg and titrating up every week only if needed) should be used for 4-6 weeks to facilitate the habit creation along with continuous sleep hygiene and behavioral corrections.
- in cases when these techniques do not help, referral to a Sleep Medicine Consultant may be considered.
- Promote early morning exposure to sunlight and physical activity during the day.
- Avoiding late physical activity, night snacks, late dinner.
- Turn off screens 1-2h before bedtime (digital curfew).
- Caregiver presence and related activities at child sleep onset, as opposed to prior to lights out, can create problematic sleep onset associations that negatively impact sleep.
- In adolescence avoid social jet leg
- Sleep facilitation can be carried out by low dose melatonin
- Promote stability of the sleep/wake cycle during the week, maintain similar sleep/wake times and bedtime routines on weekdays and weekends.
